# The Potential Effect of *Rhizoma coptidis* on Polycystic Ovary Syndrome Based on Network Pharmacology and Molecular Docking

**DOI:** 10.1155/2021/5577610

**Published:** 2021-07-08

**Authors:** Liyun Duan, De Jin, Xuedong An, Yuehong Zhang, Shenghui Zhao, Rongrong Zhou, Yingying Duan, Yuqing Zhang, Xinmin Liu, Fengmei Lian

**Affiliations:** ^1^Department of Endocrinology, Guang'anmen Hospital, China Academy of Chinese Medical Sciences, Beijing 100053, China; ^2^China Academy of Chinese Medical Sciences, Beijing 100700, China; ^3^Beijing University of Chinese Medicine, Beijing 100029, China

## Abstract

**Background:**

*Rhizoma coptidis* (RC) showed a significant effect on PCOS, but its mechanism in PCOS remains unclear.

**Methods:**

The components of RC were searched by TCMSP. The Smiles number of the active ingredients was queried through PubChem, and the predicted targets were obtained from the SwissTargetPrediction database. The DrugBank, GeneCards, and DisGeNET databases were retrieved to acquire the related targets of PCOS. Then, the network of compound-target was constructed. The core targets were analyzed using protein-protein interaction (PPI) analysis, and the binding activities were verified by molecular docking. The enriched pathways of key targets were examined by GO and KEGG.

**Results:**

13 components and 250 targets of RC on PCOS were screened. The core network was filtered based on topological parameters, and the key components were palmatine, berberine, berberrubine, quercetin, and epiberberine. The key targets included DRD2, SLC6A4, CDK2, DPP4, ESR1, AKT2, PGR, and AKT1. Molecular docking displayed that the active ingredients of RC had good binding activities with potential targets of PCOS. After enrichment analysis, 30 functional pathways were obtained, including neuroactive ligand-receptor interaction, dopaminergic synapse, and cAMP signaling pathway.

**Conclusion:**

In summary, this study clarified the potential effect of RC on PCOS, which is helpful to provide references for clinical practice. It is also conducive to the secondary development of RC and its monomer components.

## 1. Introduction

Polycystic ovary syndrome (PCOS) is an endocrine and metabolic disease in premenopausal women [1]. Depending on the diagnostic criteria, the prevalence was 4% to 21% [2], its etiology and pathogenesis are not clear, it may be related to genetic, environmental, and other factors [3], and the main manifestations are clinical and/or biochemical hyperandrogen abnormalities in ovulation and polycystic changes of the ovary [4]. PCOS is often related to insulin resistance, obesity, and dyslipidemia. PCOS increases the risk of infertility, endometrial dysfunction, cardiovascular disease, diabetes, metabolic syndrome, and other diseases [5, 6]; it seriously affects patients' physical and mental health.

At present, the therapies of PCOS are mostly symptomatic, the clinical use of oral contraceptives, antiandrogen, induction of ovulation, insulin sensitizer, and other drugs [6, 7]. However, PCOS is a multisystem disease with complex pathological mechanism, heterogeneous symptoms, and multiple complications. For single-target drugs sometimes cannot achieve good therapeutic effect, it needs to explore more targets for drugs, in order to take into account the instructions of medicine. Traditional Chinese medicine (TCM) has become a new research hotspot because of its multicomponent and multitarget characteristics. But its mechanism is complex. Based on the interaction of systematic network, it will be helpful to clarify the complex mechanism of TCM in treating diseases.

*Rhizoma coptidis* (RC), a buttercup plant, has been used for thousands of years to clear heat, dry moisture, purge fire, and detoxification [8]. It has been reported that the extracts and compounds of RC have the effects of improving insulin resistance and regulating glycolipid metabolism, antiobesity, and myocardial protection. And they have potential therapeutic effects on many kinds of diseases, for instance, PCOS, diabetes, obesity, hyperlipidemia, and cardiovascular disease [9–11]. But the potential molecular mechanism of RC has not been entirely elucidated. Research on the treatment of PCOS by RC is limited. Thus, the purpose of this study was to illustrate the potential bioactive components of RC and its mechanism of action on PCOS.

Network pharmacology is considered as a promising approach to understand compounds of TCM and predict drug or specific disease targets. It has the characteristics of comprehensive, systematic, and holistic concept, which is consistent with the traits of multicompound, multitarget, and multipathway in TCM. The use of network pharmacology in TCM is conducive to the transformation from empirical medicine to evidence-based medicine and provides multidimensional research strategies for complex TCM [12].

**Figure 1:**
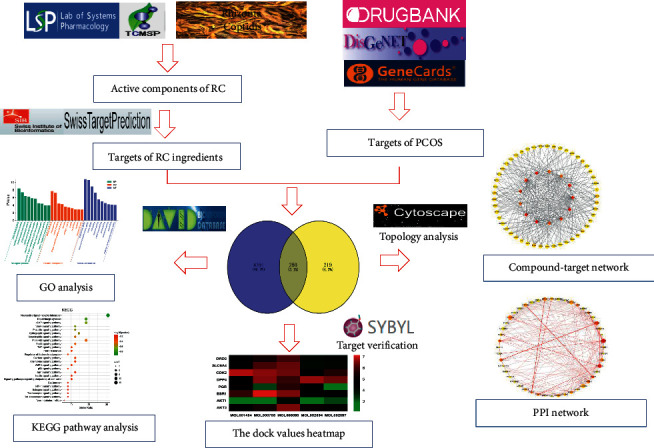
The workflow of the current network pharmacology study.

In this paper, systematic pharmacology was used to find the main constituents of RC on PCOS, to select the core targets, and to clarify the mechanisms of related pathways. Firstly, the ingredients of RC were screened from TCMSP, and the targets were predicted through the SwissTargetPrediction. Relevant targets of PCOS were achieved by searching databases. The intersection targets of RC and PCOS were obtained. Next, the interaction network of compound-target was constructed; the core network was filtered based on topology parameters. Then, the core targets were analyzed by PPI analysis, and the binding activities were verified by molecular docking. The key targets annotation and enriched pathways were examined by GO and KEGG, to study the bioactive constituents and potential mechanism of RC on PCOS. The workflow is summarized in Figure 1.

## 2. Materials and Methods

### 2.1. Screening Active Ingredients and Predicting Compounds' Targets of RC

The ingredients of RC were gathered from TCMSP (http://tcmspw.com/tcmsp.php) based on the parameters related to drug metabolism kinetics; oral bioavailability (OB) ≥30% and drug similarity (DL) ≥ 0.18 were the selecting conditions [13]. Their PubChem ID, smiles number, and 2D chemical structures could be obtained on PubChem (https://pubchem.ncbi.nlm.nih.gov/) [14]. The targets were predicted through the SwissTargetPrediction database (http://www.swisstargetprediction.ch/) [15], and they were imported into UniProt (http://www.uniprot.org/) for batch standardization [16], setting the organism “Homo sapiens.”

### 2.2. Collecting Targets of PCOS

The PCOS targets were searched in the DrugBank (https://www.drugbank.ca/) [17], DisGeNET (https://www.disgenet.org/) [18], and GeneCards platform (https://www.genecards.org/) [19] with the keywords “Polycystic ovary syndrome” and “Polycystic Ovarian Syndrome.” Then the targets were merged and duplication was gotten rid of.

### 2.3. Compound-Target Network

The targets of intersection between RC and PCOS were taken to establish the network diagram using Cytoscape 3.7.2. The topological properties of network were analyzed. To clarify the mechanism of RC on PCOS better, the core network was selected based on topological parameters. The degree and the betweenness centrality were key indexes to estimate the importance of nodes. As the degree and betweenness centrality increase, the nodes are more important in the network.

### 2.4. Protein-Protein Interaction Analysis

The core targets were filtered based on topological analysis, to achieve the interaction among the targets. These targets were input into the String database (https://string-db.org/) [20]. The results of the visual analysis were acquired by Cytoscape 3.7.2. Cytoscape-MCODE was applied to cluster analysis and Cytoscape-hubba was used for screening hub targets.

### 2.5. Molecular Docking Verification

The binding activities were verified by molecular docking to illustrate the relationship between the key active compounds and the proteins of targets. According to Cytoscape-hubba and related literatures, the genes of targets closely related to PCOS were selected, and the corresponding proteins were obtained in Protein Data Bank (PDB) (http://www.rcsb.org/pdb/home/home.do) [21]. The selected proteins were corresponded to the UniProt number of the intersecting targets of RC and PCOS. Then the crystal structures of screened targets were embellished by the SYBYL-X (version 2.1.1) software; removing the ligands, adding hydrogen, removing water, optimizing, and patching amino acids were involved. Then the Surflex-Dock module was used for molecular docking. The total score was used as the standard for evaluation of docking results. The binding activities become stronger as the total score increases.

### 2.6. GO and KEGG Pathway Enrichment Analysis

David database (https://david.ncifcrf.gov/summary.jsp) is a tool for gene annotation, enrichment, and pathway analysis [22]. The GO and KEGG pathway enrichment analysis were used to examine the key targets [23]. GO terms with *P* values <0.01 were employed.

## 3. Results

### 3.1. Active Compounds and Related Targets

Through the TCMSP database, 48 compounds of RC were obtained. Selecting the compounds OB ≥ 30% and DL ≥ 0.18, 14 active compounds were acquired, mainly including palmatine, berberine, berberrubine, (R)-canadine, epiberberine, and others. Then 1000 predicted targets were obtained in the SwissTargetPrediction database. Calibration and deweighting by the UniProt database, under the condition of “Homo sapiens,” 469 targets were obtained. A total of 4641 targets of PCOS were achieved from the disease target databases. After the intersection of targets among RC and PCOS, 13 compounds and 250 targets were obtained. The 13 compounds are shown in additional file 1: Table S1. The 250 targets are listed in additional file 2: Table S2.

### 3.2. The Network of Compound-Target

**Figure 2:**
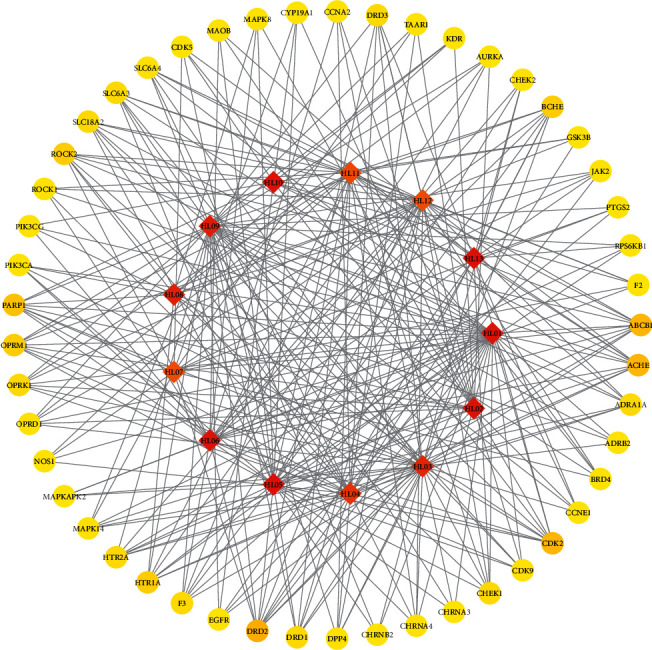
The network of the core network.

The network of compound-targets and topological analysis were made through Cytoscape 3.7.2. The degree values of compound-targets were computed. The degree is positively correlated with the relationship between compounds and targets. According to the degree of a topological analysis, from the average degree (4.616) to the maximum degree (67), 49 targets were screened out, which are listed in Table 1. These 49 core targets were built in the core network, as shown in Figure 2. The compounds were represented by diamond node, and the targets of PCOS were represented by ellipse node. The color of node changing from yellow to red is equivalent to the degree from small to large. The numerical label represents the compound, as shown in additional file 1: Table S1. The target labels are represented by the target symbols. Based on topological parameters, the top five-degree compounds of RC were palmatine, berberine, berberrubine, (R)-Canadine, and epiberberine.

### 3.3. Protein-Protein Interaction Network

**Figure 3:**
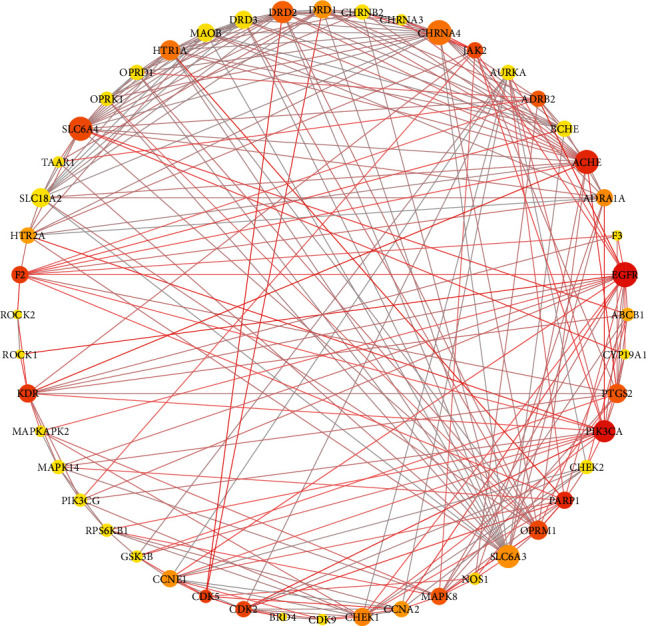
The interaction relationship between core targets.

The String database was used to explore the interaction relationship between core targets. The results are described in Figure 3. Circle nodes represent the targets. The size of the node is positively correlated with the degree. As the betweenness centrality increases, the color of the node changed from yellow to red. The degree and the betweenness centrality represents the importance of the targets. The thickness of line and the color depth of the nodes change with the size of the edge betweenness value. The brighter the color and the thicker of the connection line between the nodes, the closer the interaction relationship between the targets. Meanwhile, the module analysis of PPI network was conducted by MCODE of Cytoscape 3.7.2. to identify the highly interacting nodes. These clusters were screened based on default parameters of nodes and score values which are shown in Table 2. Then, different algorithms were used to screen the hub genes, as shown in Table 3. DRD2, HTR1A, SLC6A3, SLC6A4, CHRNA4, ACHE, and OPRM1 were the common key targets under different algorithms.

### 3.4. Molecular Docking Results

**Figure 4:**
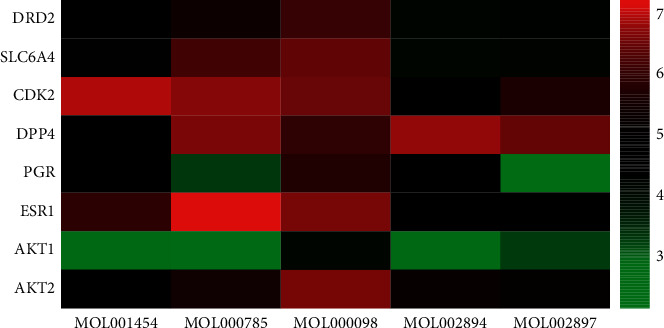
Molecular docking results.

Based on topological parameters and related literature analysis, the active compounds of RC including palmatine, berberine, quercetin, berberrubine, and epiberberine were screened. Based on the common key targets under different algorithms and targets closely related to PCOS, the key targets of DRD2, SLC6A4, CDK2, DPP4, ESR1, AKT2, AKT1, and PGR were selected. Then the active compounds of RC and the key targets were verified by SYBYL-X 2.1.1 software. The total score was used as the evaluation standard for the docking result analysis. The molecular docking score is supplied in additional file 3: Table S3. The results showed that both targets and the compounds could bind well; the RC active ingredients had good binding activities with potential targets, as shown in Figure 4. Furthermore, based on the total score, the targets ranking from high to low were CDK2, DPP4, ESR1, AKT2, SLC6A4, DRD2, PGR, and AKT1. CDK2, DPP4, and ESR1 may be the key blinding ligands of targets concerned with the therapeutic effect of PCOS. The five compounds ranking from high to low were quercetin, palmatine, berberine, berberrubine, and epiberberine. It indicates that quercetin, palmatine, and berberine are more likely to bind to the core targets of the treatment of PCOS.

### 3.5. GO Enrichment Analysis

**Figure 5:**
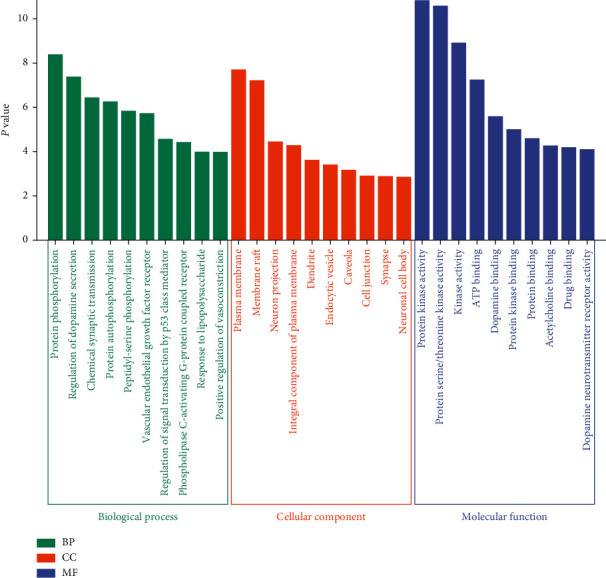
GO enrichment analysis of key targets.

DAVID was used for GO enrichment analysis of key targets, and 131 GO terms with *P* values <0.01 were acquired, including 87 entries of biological process (BP), 21 entries of cell composition (CC), and 23 entries of molecular function (MF). The GO terms closely related to the disease were screened out. The top 10 enriched GO terms are listed in Figure 5. In terms of biological process, these targets were mainly related to protein phosphorylation, regulation of dopamine secretion, chemical synaptic transmission, protein autophosphorylation, peptidyl-serine phosphorylation, and others. In cell composition, the targets were associated with the plasma membrane, membrane raft, neuron projection, integral component of the plasma membrane, dendrite, endocytic vesicle, caveola, and so on. In molecular function, the targets were concerned with protein kinase activity, protein serine/threonine kinase activity, kinase activity, ATP binding, dopamine binding, protein kinase binding, and protein binding.

### 3.6. KEGG Enrichment Analysis

**Figure 6:**
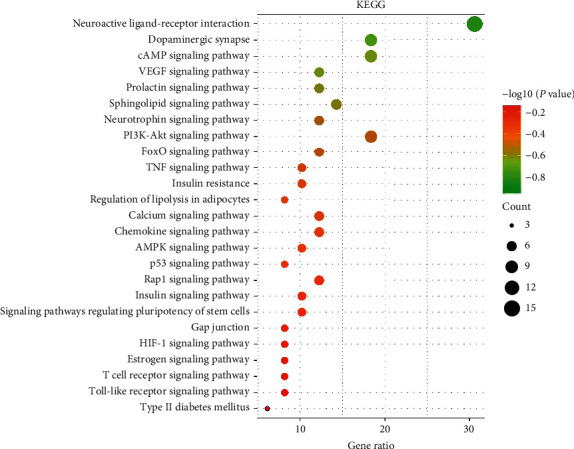
KEGG enrichment analysis.

There were 49 key targets of RC enriched in 73 signaling pathways after the KEGG enrichment analysis. Through literature investigation, 30 pathways were prominently associated with PCOS, and the related genes are listed in additional file 4: Table S4. A bubble diagram is drawn by the top 25 signal pathways based on the *P* value in Figure 6. In addition, the top 15 signaling pathways and their related genes are listed in Table 4. RC mainly treats PCOS through neuroactive ligand-receptor interaction, dopaminergic synapse, cAMP signaling pathway, PI3K-Akt signaling pathway, and so on.

## 4. Discussion

According to the assessment of pharmacokinetics and network pharmacology, 13 components of RC had a potential effect on PCOS, mainly including berberine, palmatine, quercetin, berberrubine, and epiberberine. Berberine has been found to be safe and effective in terms of improving insulin resistance (IR), increasing ovulation rate per cycle, increasing the live birth rate, lowering body weight, and improving blood lipids [24]. Berberine can treat PCOS by regulating insulin signaling pathway and increasing insulin sensitivity [25]. Berberine also has the potential to treat PCOS of rat model through upregulating GLUT4 via activating PI3K/AKT and suppressing MAPK pathway [26]. Palmatine has the effects of antioxidation, anti-inflammation, and regulating blood lipids. It can cure different diseases [27]. Regarding inflammation and oxidative stress, palmatine is a substrate for P-glycoprotein (P-gp). Studies have found that patients with insulin resistance are more likely to absorb palmatine, and the effects of palmatine on metabolic diseases need further clinical verification [28]. Quercetin has the effect of radical scavenging and antioxidant properties [29]. Studies have shown that quercetin can improve ovarian tissue; it may prevent PCOS complications by improving IR and chronic inflammation and reduce testosterone, luteinizing hormone (LH), and resistin levels, and it can increase the insulin sensitivity by promoting the proliferation of pancreatic *β*-cells [30]. Quercetin can decrease resistin plasma concentration and gene expression, reduce LH and testosterone level in overweight or obese women with PCOS, and improve insulin resistance [31]. Quercetin can reduce PCOS-IR and induce uterine GLUT4 and ER*α* gene expression in PCOS rats [32]. Quercetin treats PCOS rats model by restraining PI3K which can reduce the androgen production by inhibiting the expression of CYP17A1 gene [33]. PCOS has the detrimental inflammatory and metabolic characteristics, quercetin may improve the metabolic features of PCOS by upregulating AMPK and adiponectin receptors, and it can elevate expression of adiponectin receptors at the transcript level and enhance the AMPK level [34]. Berberrubine is deemed as one of the major active metabolites of berberine; berberrubine exerted multiple activities, including anti-inflammation, antimicrobial, lipid-lowering, and antioxidation [35]. Epiberberine has antiadipogenesis, antidyslipidemia, and other effects [9].

Based on the analysis of network, the key targets of DRD2, SLC6A4, CDK2, DPP4, ESR1, AKT2, AKT1, and PGR were selected. They might be the core targets of RC closely related to PCOS. Studies have shown that the expression of DRD2 decreased in PCOS ovaries [36]. The D2/Drd2 pathway modulates VEGF-dependent vascular permeability and angiogenesis in the ovary under physiological conditions; the D2/Drd2 system acts as a negative regulator of vascularization [37]. Decreased Drd2 expression in the theca was related to an increased vascularization in the theca of PCOS follicles, and overproduction of VEGF may lead to ovarian hyperstimulation syndrome [36, 38]. SLC6A4 5HTTLPR polymorphism was related to insulin secretion and insulin blood levels during OGTT in patients with PCOS [39]. CDK2 is a cell cycle regulator, and the PCOS condition is related to the increased nuclear abundance of CDK2 in the endometrial epithelial cells [40]. Dipeptidyl peptidase-4 (DPP4) is an adipokine related to IR. PCOS is also associated with IR [41]. Some studies found that the DPP4 inhibitors may be helpful for treating PCOS [42]. Genetic polymorphisms involved in estrogen action may contribute to women's susceptibility to PCOS, estrogen receptor alpha gene (ESR1) PvuII, and XbaI polymorphisms are associated with metabolic and proinflammatory factors in PCOS [43]. Dysfunction of miR-135a and miR-186 can lead to abnormal function of granulosa cells by targeting ESR2, leading to the occurrence of PCOS [44]. AKTs influence the proliferation of granulosa-lutein cell (GC). There is a positive correlation between the expression levels of AKT1 and AKT2 with androgens, and high expressions of AKT1 and AKT2 may cause GCs dysfunction in the PCOS patients with hyperandrogenemia and may lead to PCOS [45]. Impaired progesterone (P4) signaling can lead to complications of PCOS. Increased expression of progesterone receptor (PGR) isoforms A and B parallel elevated estrogen receptor (ER) expression in PCOS-like rat uteri [46]. In addition, HTR1A, SLC6A3, SLC18A2, MMP9, TNF, AR, and other targets are also closely related to PCOS through literature analysis. The function of HTR1A can stimulate a biological process which is relevant to the regulation of cell growth and cell survival [47]. SLC6A3 was related to BMI. The studies found that the allele 10 of the VNTR in the SLC6A3 is decreased with a low expression of the dopamine transporter [48]. SLC18A2 is crucial in dopamine regulation. Studies have found that genetic variants in SLC18A2 were associated with FSH levels; it might take part in follicular development and maturation in PCOS [49]. Matrix metalloproteinases (MMPs) play a key role in the pathogenesis of PCOS. Gelatinase activity of MMP9 and MMP9/TIMP1 ratio were found significantly higher in PCOS, and MMP9 may be related to the pathogenesis of PCOS [50]. PCOS is related to low-grade chronic inflammation and insulin resistance mediated by proinflammatory cytokines. TNF level, TNF/IL-6, TNF/IL-2, and TNF/IL-4 ratios were found decreased, and these changes may be helpful to intervene patients with PCOS [51]. Androgens play an important role in regulating female fertility. Considerable evidences suggest that androgens in the excess act through AR and play an important in the origins of PCOS [52]. In a word, these key targets were important to treat PCOS.

PCOS is a complex endocrine system disease in women of reproductive age [53], which involved different metabolic pathways. According to KEGG enrichment analysis of key targets and relevant literature, 30 metabolic pathways closely related to disease were obtained. The top 10 signaling pathways were described. Neuroactive ligand-receptor interaction: clinical studies have found that the changes in endometrial gene expression in patients with PCOS are associated with neuroactive ligand-receptor interaction [54]. Dopaminergic synapse: dopamine receptor 2 agonists may be used to reduce the risk of ovarian hyperstimulation syndrome. The lutein granulosa cells of PCOS treated with cabergoline showed more significant changes than non-PCOS, and dopaminergic synapse was highlighted among the prioritized functions [55]. Dopamine can negatively regulate the level of FSH and insulin secretion through the D2 receptor [49]. cAMP signaling pathway: the cAMP signaling regulates the excitability of gonadotropin-releasing hormone (GnRH) neurons [56]. GnRH regulates the level of female reproductive hormones, such as luteinizing hormone, and affects ovulation. Irregular expression of CYP19A1 is participated in the progress of PCOS. In human ovarian granulosa cells, activation of the cAMP/protein kinase A/cAMP response element-binding protein pathway plays an important role in FSH regulation of CYP19A1 [57]. VEGF signaling pathway: VEGF is related to angiogenesis in the ovary. HIF-1a/VEGF signaling pathway can regulate the development of ovarian luteum and may provide a new strategy for ovarian dysfunction diseases such as PCOS [58]. Prolactin signaling pathway: prolactin is an important hormone that affects reproduction, prolactin signaling, and its short isoform receptors also have an effect on ovarian follicles [59]. Sphingolipid signaling pathway: there is some evidence that intracellular lipids may contribute to insulin resistance by inhibiting insulin signaling, and the intracellular lipid pools are related to the sphingomyelin signaling pathway [60]. Neurotrophin signaling pathway: neurotrophin and neurotrophic factor signaling events have substantial roles in the ovary. Dysregulation of the neurotrophin system may negatively affect ovarian function, leading to PCOS [61]. PI3K-Akt signaling pathway: the alterations of PI3K/Akt signaling pathway were involved in the progression of PCOS-IR. It can reduce insulin resistance by acting on the PI3K/Akt signaling pathway [62]. FoxO signaling pathway: high level of FoxO 1 signaling correlated with the production of proinflammatory cytokines, and the FoxO FoxO 1 knockdown restrained the glucose uptake in PCOS macrophages [63]. TNF signaling pathway: PCOS is associated with chronic inflammation, and anti-TNF-*α* therapy may reduce excessive androgen levels, which may alleviate the symptoms of PCOS [64]. In addition to the above signaling pathways, insulin resistance, AMPK signaling pathway, HIF-1 signaling pathway, mTOR signaling pathway and other metabolic pathways were also closely related to PCOS. Insulin resistance: improving insulin resistance can promote ovulation and regular menstruation [65]. AMPK signaling pathway: it can improve insulin resistance and inhibit apoptosis in PCOS mice by activating the AMPK/PI3K/AKT/FoxO3a signaling pathway [66]. By means of activating the AMPK/mTOR/Akt signaling pathway, the expression of MMP2 and MMP9 can be decreased, the severity of PCOS can be alleviated, and normal ovulation can be promoted [67]. HIF-1 signaling pathway: HIF-1a signaling is important in PCOS, and activation of HIF-1a/ET-2 signaling pathway can improve PCOS [68]. mTOR signaling pathway: inhibitors of the mTOR signaling pathway can restrain cell proliferation, differentiation, and upregulation of protein expression, and it can effectively treat PCOS [69]. Studies have shown that the mTOR signaling system is a key pathophysiological basis of PCOS; its overexpression can result in insulin resistance and influence the growth of follicles directly [70].

The study clarified the multicomponent and multitarget of RC in the therapy of PCOS. After KEGG analysis, neuroactive ligand-receptor interaction was the most enriched signaling pathway. Dopaminergic synapse, cAMP signaling pathway, and PI3K-Akt signaling pathway were also associated with PCOS closely. This indicated that these signaling pathways might be the critical pathways in treating PCOS. Moreover, there were 13 ingredients of RC which were selected out, and the 5 key ingredients were testified by molecular docking. But this study has some limitations on account of the fact that results were based just on data mining and network pharmacological analysis. The ingredients with better pharmacokinetic parameters of RC were studied, but some unidentified components were still not took in. The mechanism of RC in the therapy of PCOS still needs further evaluation.

## 5. Conclusion

This study explored the potential bioactive ingredients of RC and the mechanism of RC on PCOS according to network pharmacology. The network was built including 13 key ingredients and 49 core targets. Furthermore, the five key active ingredients were screened, including palmatine, berberine, berberrubine, quercetin, and epiberberine. The interaction between the key cores was also studied. The molecular docking testified that the active ingredients of RC had good activity against PCOS targets. In the end, the results indicated that 30 functional pathways of RC took part in the therapy of PCOS by means of data mining and network analysis. The effect of RC on PCOS has the characteristics of multitarget and multipathway. This study is helpful to provide references for clinical practice, and it is also conducive to the secondary development of RC and its monomer components, but the mechanism still needs to be further explored based on experimental study.

## Figures and Tables

**Table 1:** The information of 49 core targets ranked by degree.

Target symbol	UniProt ID	Target symbol	UniProt ID	Target symbol	UniProt ID
DRD2	P14416	HTR2A	P28223	MAOB	P27338
ABCB1	P08183	OPRD1	P41143	MAPK8	P45983
ACHE	P22303	SLC6A4	P31645	CYP19A1	P11511
CDK2	P24941	CDK9	P50750	CCNA2	P20248
PARP1	P09874	CHRNA4	P43681	TAAR1	Q96RJ0
OPRM1	P35372	DPP4	P27487	KDR	P35968
ROCK2	O75116	MAPK14	Q16539	CHEK2	O96017
BCHE	P06276	PIK3CA	P42336	GSK3B	P49841
HTR1A	P08908	ROCK1	Q13464	PTGS2	P35354
OPRK1	P41145	CDK5	Q00535	ADRB2	P07550
SLC18A2	Q05940	AURKA	O14965	RPS6KB1	P23443
SLC6A3	Q01959	JAK2	O60674	CHRNA3	P32297
DRD3	P35462	BRD4	O60885	CHRNB2	P17787
ADRA1A	P35348	CCNE1	P24864	MAPKAPK2	P49137
CHEK1	O14757	EGFR	P00533	NOS1	P29475
DRD1	P21728	F2	P00734		
F3	P13726	PIK3CG	P48736		

**Table 2:** List of clusters from MCODE.

Cluster	Score (density)	Nodes	Edges	Node IDs
1	8	9	32	ACHE, SLC18A2, OPRD1, OPRK1, DRD3, HTR1A, DRD2, CHRNA4, OPRM1
2	5.077	14	33	BCHE, AURKA, PARP1, CCNE1, CHEK1, SLC6A4, CCNA2, SLC6A3, ADRA1A, HTR2A, CHRNB2, DRD1, MAOB, PIK3CA
3	3.5	5	7	ABCB1, CYP19A1, EGFR, PTGS2, F3

**Table 3:** The top 10 hub genes ranked with different algorithms.

Category	Rank method in CytoHubba				
	MCC	MNC	Degree	EPC	Closeness
Gene symbol top 10	CHRNA4	CHRNA4	CHRNA4	CHRNA4	EGFR
	DRD2	EGFR	EGFR	ACHE	ACHE
	HTR1A	ACHE	ACHE	SLC6A4	CHRNA4
	DRD3	SLC6A3	SLC6A4	SLC6A3	PIK3CA
	ACHE	SLC6A4	SLC6A3	HTR1A	SLC6A4
	SLC18A2	PIK3CA	PIK3CA	DRD2	DRD2
	OPRM1	DRD2	DRD2	SLC18A2	PTGS2
	SLC6A3	HTR1A	HTR1A	MAOB	SLC6A3
	SLC6A4	SLC18A2	SLC18A2	EGFR	HTR1A
	MAOB	OPRM1	OPRM1	OPRM1	OPRM1

**Table 4:** The top 15 signaling pathways with related genes.

Term	Pathway	Genes
hsa04080	Neuroactive ligand-receptor interaction	CHRNB2, OPRD1, CHRNA3, CHRNA4, HTR1A, OPRK1, ADRB2, HTR2A, OPRM1, F2, TAAR1, ADRA1A, DRD1, DRD2, DRD3
hsa04728	Dopaminergic synapse	GSK3B, MAPK8, MAOB, DRD1, DRD2, MAPK14, DRD3, SLC6A3, SLC18A2
hsa04024	cAMP signaling pathway	MAPK8, PIK3CA, ROCK1, ROCK2, HTR1A, DRD1, ADRB2, DRD2, PIK3CG
hsa04370	VEGF signaling pathway	PIK3CA, MAPKAPK2, KDR, MAPK14, PTGS2, PIK3CG
hsa04917	Prolactin signaling pathway	GSK3B, MAPK8, PIK3CA, JAK2, MAPK14, PIK3CG
hsa04071	Sphingolipid signaling pathway	OPRD1, MAPK8, PIK3CA, ROCK1, ROCK2, MAPK14, PIK3CG
hsa04722	Neurotrophin signaling pathway	GSK3B, MAPK8, PIK3CA, MAPKAPK2, MAPK14, PIK3CG
hsa04151	PI3K-Akt signaling pathway	GSK3B, RPS6KB1, PIK3CA, CCNE1, CDK2, KDR, JAK2, EGFR, PIK3CG
hsa04068	FoxO signaling pathway	MAPK8, PIK3CA, CDK2, MAPK14, EGFR, PIK3CG
hsa04668	TNF signaling pathway	MAPK8, PIK3CA, MAPK14, PTGS2, PIK3CG
hsa04931	Insulin resistance	GSK3B, MAPK8, RPS6KB1, PIK3CA, PIK3CG
hsa04923	Regulation of lipolysis in adipocytes	PIK3CA, ADRB2, PTGS2, PIK3CG
hsa04020	Calcium signaling pathway	DRD1, ADRB2, NOS1, HTR2A, ADRA1A, EGFR
hsa04062	Chemokine signaling pathway	GSK3B, PIK3CA, ROCK1, ROCK2, JAK2, PIK3CG
hsa04152	AMPK signaling pathway	CCNA2, RPS6KB1, PIK3CA, ADRA1A, PIK3CG

## Supplementary Materials

Supplementary Materials Additional file 1: Table S1: the active compounds of RC. Additional file 2: Table S2: the intersection targets related to RC and PCOS. Additional file 3: Table S3: the molecular docking score. Additional file 4: Table S4: the detailed information about 30 related pathways.
