# Supplementary material for: The Potential Effect of Rhizoma coptidis on Polycystic Ovary Syndrome Based on Network Pharmacology and Molecular Docking
Source: Evid Based Complement Alternat Med. 2021 Jul 8;2021:5577610. doi: 10.1155/2021/5577610 (PMC8282388; doi:10.1155/2021/5577610)
Supplement: Supplementary Materials — Additional file 1: Table S1: the active compounds of RC. Additional file 2: Table S2: the intersection targets related to RC and PCOS. Additional file 3: Table S3: the molecular docking score. Additional file 4: Table S4: the detailed information about 30 related pathways. [file 5577610.f1.docx]

**Supplementary information**

**Additional file 1: Table S1.**

**The active compounds of RC.**

| **Mol ID** | **No** | **Compound** | **OB** | **DL** | **SMILES** |
| --- | --- | --- | --- | --- | --- |
| MOL001454 | HL01 | berberine | 36.86 | 0.78 | O1c2cc3CCn4c(c3cc2OC1)cc1c(c4)c(OC)c(OC)cc1 |
| MOL013352 | HL02 | Obacunone | 43.29 | 0.77 | O1[C@@]23[C@]4([C@@H]([C@@]5([C@@H](CC4=O)C(OC(=O)C=C5)(C)C)C)CC[C@]2([C@@H](OC(=O)[C@@H]13)c1ccoc1)C)C |
| MOL002894 | HL03 | berberrubine | 35.74 | 0.73 | O1c2cc3CCn4c(c3cc2OC1)cc1c(c4)c(O)c(OC)cc1 |
| MOL002897 | HL04 | epiberberine | 43.09 | 0.78 | O1c2c3cn4CCc5c(c4cc3ccc2OC1)cc(OC)c(OC)c5 |
| MOL002903 | HL05 | (R)-Canadine | 55.37 | 0.77 | c12c(c(c(cc1)OC)OC)CN1[C@H](C2)c2c(CC1)cc1c(c2)OCO1 |
| MOL002904 | HL06 | Berlambine | 36.68 | 0.82 | c12c3c(cc4c(c3)OCO4)CCn1c(=O)c1c(c2)ccc(c1OC)OC |
| MOL000622 | HL07 | Magnograndiolide | 63.71 | 0.19 | [C@@H]12[C@@H]([C@@H]3[C@@H](CC[C@@]1(C)O)C(=C)C(=O)O3)[C@@](CC2)(O)C |
| MOL000762 | HL08 | Palmidin A | 35.36 | 0.65 | c12c([C@H](c3c(C1=O)c(cc(c3)C)O)[C@H]1c3c(C(=O)c4c1cc(cc4O)CO)c(ccc3)O)cc(cc2O)O |
| MOL000785 | HL09 | palmatine | 64.6 | 0.65 | c12c3c(cc(c(c3)OC)OC)CCn1cc1c(c2)ccc(c1OC)OC |
| MOL000098 | HL10 | quercetin | 46.43 | 0.28 | c1(cc(c2c(c1)oc(c(c2=O)O)c1ccc(c(c1)O)O)O)O |
| MOL001458 | HL11 | coptisine | 30.67 | 0.86 | O1c2cc3c4n(CCc3cc2OC1)cc1c(c4)ccc2OCOc12 |
| MOL002668 | HL12 | Worenine | 45.83 | 0.87 | O1c2cc3c4n(CCc3cc2OC1)cc1c(c4C)cc2OCOc2c1 |
| MOL008647 | HL13 | Moupinamide | 86.71 | 0.26 | O(c1cc(/C=C/C(=O)NCCc2ccc(O)cc2)ccc1O)C |

**Additional file 2: Table S2.**

**The intersection targets related to RC and PCOS.**

| **Target symbol** | **Uniprot ID** | **Target symbol** | **Uniprot ID** | **Target symbol** | **Uniprot ID** | **Target symbol** | **Uniprot ID** |
| --- | --- | --- | --- | --- | --- | --- | --- |
| PGR | P06401 | CNR1 | P21554 | MAPK1 | P28482 | PDE4D | Q08499 |
| NR3C2 | P08235 | ROS1 | P08922 | BRAF | P15056 | SCN1A | P35498 |
| AR | P10275 | FAAH | O00519 | CHEK2 | O96017 | PTGS1 | P23219 |
| NR3C1 | P04150 | HSD11B2 | P80365 | IGF1R | P08069 | PRKDC | P78527 |
| ESR1 | P03372 | EGFR | P00533 | ERBB2 | P04626 | MAOA | P21397 |
| CYP19A1 | P11511 | WNT3A | P56704 | SRC | P12931 | HDAC1 | Q13547 |
| ABCB1 | P08183 | PIK3R1 | P27986 | AVPR2 | P30518 | DYRK1A | Q13627 |
| SHBG | P04278 | TGFBR1 | P36897 | CCND1 | P24385 | HDAC6 | Q9UBN7 |
| AKR1C3 | P42330 | PTGS2 | P35354 | MET | P08581 | ABCC9 | O60706 |
| MMP2 | P08253 | MTNR1A | P48039 | ATR | Q13535 | PRKCA | P17252 |
| MTNR1B | P49286 | AGTR1 | P30556 | NEK1 | Q96PY6 | SLC6A3 | Q01959 |
| AKT2 | P31751 | EPHX1 | P07099 | KIT | P10721 | MMP12 | P39900 |
| AKR1C1 | Q04828 | CYP1B1 | Q16678 | PRKD1 | Q15139 | CTSB | P07858 |
| CCNB1 | P14635 | AKR1B1 | P15121 | PLAU | P00749 | ABCC1 | P33527 |
| AKR1C2 | P52895 | F3 | P13726 | ABL1 | P00519 | PTK2 | Q05397 |
| TOP2A | P11388 | SLC47A2 | Q86VL8 | TTR | P02766 | ILK | Q13418 |
| TTK | P33981 | DAPK1 | P53355 | PDE11A | Q9HCR9 | ABCG2 | Q9UNQ0 |
| CAMK2D | Q13557 | TOP1 | P11387 | CHEK1 | O14757 | SYK | P43405 |
| ADRB2 | P07550 | CYP11B1 | P15538 | PTPN1 | P18031 | HTR2A | P28223 |
| ADRB3 | P13945 | ADRA2B | P18089 | CYP11B2 | P19099 | ANPEP | P15144 |
| INSR | P06213 | F2 | P00734 | CDK4 | P11802 | CCNE1 | P24864 |
| ADRA1D | P25100 | SLC6A2 | P23975 | BTK | Q06187 | AVPR1A | P37288 |
| ADRA1A | P35348 | SLC18A2 | Q05940 | GABRA1 | P14867 | CCNA2 | P20248 |
| ADRA1B | P35368 | DHCR7 | Q9UBM7 | GLI2 | P10070 | TAAR1 | Q96RJ0 |
| LIPE | Q05469 | DRD3 | P35462 | TNNT2 | P45379 | HTR1A | P08908 |
| CDK2 | P24941 | EPHB2 | P29323 | CSF1R | P07333 | SLC6A4 | P31645 |
| BRD2 | P25440 | EPHA7 | Q15375 | APP | P05067 | AKR1C4 | P17516 |
| FTO | Q9C0B1 | ROCK2 | O75116 | KDR | P35968 | MMP13 | P45452 |
| TNF | P01375 | NAMPT | P43490 | PSMB8 | P28062 | RPS6KA1 | Q15418 |
| LIMK1 | P53667 | CPT1A | P50416 | PSEN1 | P49768 | PDE5A | O76074 |
| VDR | P11473 | MMP1 | P03956 | PRKACA | P17612 | PER2 | O15055 |
| MMP14 | P50281 | MPG | P29372 | AURKA | O14965 | ACHE | P22303 |
| DPP4 | P27487 | MPO | P05164 | IMPDH1 | P20839 | LIMK2 | P53671 |
| HSD11B1 | P28845 | NOS2 | P35228 | PARP1 | P09874 | ERAP2 | Q6P179 |
| ESR2 | Q92731 | HSP90AA1 | P07900 | DRD2 | P14416 | ADAMTS4 | O75173 |
| PLG | P00747 | PDK1 | Q15118 | MAPK14 | Q16539 | SCN9A | Q15858 |
| PIK3CG | P48736 | HTR2C | P28335 | GSTP1 | P09211 | FAP | Q12884 |
| PIK3CD | O00329 | MDM2 | Q00987 | HDAC4 | P56524 | CYP2D6 | P10635 |
| PIK3CB | P42338 | JUN | P05412 | TYR | P14679 | OPRM1 | P35372 |
| PIK3CA | P42336 | JAK2 | O60674 | MMP7 | P09237 | MAPK10 | P53779 |
| MMP9 | P14780 | ITK | Q08881 | SELP | P16109 | MAPKAPK2 | P49137 |
| MAPK3 | P27361 | MIF | P14174 | SERPINA6 | P08185 | DRD5 | P21918 |
| PDCD4 | Q53EL6 | GCK | P35557 | PSEN2 | P49810 | ACVRL1 | P37023 |
| AKT1 | P31749 | ROCK1 | Q13464 | ALK | Q9UM73 | MMP8 | P22894 |
| MTOR | P42345 | RPS6KB1 | P23443 | AXL | P30530 | DNM1 | Q05193 |
| GSK3B | P49841 | RAC1 | P63000 | XDH | P47989 | PGK1 | P00558 |
| HSD17B1 | P14061 | APEX1 | P27695 | SLC22A12 | Q96S37 | CRHR1 | P34998 |
| HSD17B2 | P37059 | HNF4A | P41235 | FBP1 | P09467 | OPRK1 | P41145 |
| KCNJ11 | Q14654 | HMGCR | P04035 | KCNJ1 | P48048 | EIF2AK3 | Q9NZJ5 |
| MAPK8 | P45983 | HPGDS | O60760 | TNNI3 | P19429 | DRD1 | P21728 |
| HTR2B | P41595 | NOS1 | P29475 | CAPN1 | P07384 | CHRNB3 | Q05901 |
| CTSK | P43235 | CHRNA4 | P43681 | EPHX2 | P34913 | GABRA2 | P47869 |
| CDK5 | Q00535 | CXCR2 | P25025 | BRD4 | O60885 | GPR35 | Q9HC97 |
| CSNK1D | P48730 | OPRD1 | P41143 | NEK6 | Q9HC98 | CHRNA6 | Q15825 |
| RXRA | P19793 | BAZ2A | Q9UIF9 | CHRNA3 | P32297 | PTPRS | Q13332 |
| MERTK | Q12866 | MAP3K14 | Q99558 | CA4 | P22748 | DNPEP | Q9ULA0 |
| BCHE | P06276 | P2RX7 | Q99572 | TRAP1 | Q12931 | MAP4K4 | O95819 |
| MAOB | P27338 | CHRNB2 | P17787 | CHRNA2 | Q15822 | EPHA5 | P54756 |
| HSP90B1 | P14625 | CXCR1 | P25024 | CDK9 | P50750 | CLK1 | P49759 |
| HTR6 | P50406 | PRKCZ | Q05513 | CFD | P00746 | CA7 | P43166 |
| IKBKB | O14920 | PNMT | P11086 | EPHA4 | P54764 | CDK6 | Q00534 |
| BMP1 | P13497 | ADAM17 | P78536 | TRPM8 | Q7Z2W7 |  |  |
| FDFT1 | P37268 | NEK2 | P51955 | CHRNB4 | P30926 |  |  |

**Additional file 3: Table S3.**

**The Molecular docking score.**

| **S3 Molecular docking score** | | | | | | |
| --- | --- | --- | --- | --- | --- | --- |
| **PDB(ID)** | **MOL001454** | **MOL000785** | **MOL000098** | **MOL002894** | **MOL002897** | **Gene name** |
| 6CM4 | 4.4751 | 5.3099 | 5.9967 | 4.156 | 4.2778 | DRD2 |
| 5I6Z | 4.3954 | 6.0962 | 6.3769 | 4.1029 | 4.2175 | SLC6A4 |
| 1B39 | 6.8742 | 6.6342 | 6.4306 | 4.8999 | 5.6213 | CDK2 |
| 4L72 | 4.7215 | 6.572 | 5.9066 | 6.7138 | 6.3919 | DPP4 |
| 1A28 | 4.6945 | 3.2538 | 5.6962 | 4.4287 | 2.1095 | PGR |
| 4XI3 | 5.861 | 7.1502 | 6.5379 | 4.9206 | 4.8212 | ESR1 |
| 2UZR | 2.431 | 2.2796 | 4.1345 | 2.5229 | 3.24 | AKT1 |
| 1MRY | 4.5878 | 5.3828 | 6.5319 | 5.1361 | 5.0246 | AKT2 |

**Additional file 4: Table S4.**

**The detailed information about 30 related pathways.**

| **Term** | **Count** | **%** | **PValue** | **Genes** |
| --- | --- | --- | --- | --- |
| hsa04080:Neuroactive ligand-receptor interaction | 15 | 30.6122449 | 1.60E-09 | CHRNB2, OPRD1, CHRNA3, CHRNA4, HTR1A, OPRK1, ADRB2, HTR2A, OPRM1, F2, TAAR1, ADRA1A, DRD1, DRD2, DRD3 |
| hsa04728:Dopaminergic synapse | 9 | 18.3673469 | 1.66E-06 | GSK3B, MAPK8, MAOB, DRD1, DRD2, MAPK14, DRD3, SLC6A3, SLC18A2 |
| hsa04024:cAMP signaling pathway | 9 | 18.3673469 | 4.17E-05 | MAPK8, PIK3CA, ROCK1, ROCK2, HTR1A, DRD1, ADRB2, DRD2, PIK3CG |
| hsa04370:VEGF signaling pathway | 6 | 12.244898 | 4.81E-05 | PIK3CA, MAPKAPK2, KDR, MAPK14, PTGS2, PIK3CG |
| hsa04917:Prolactin signaling pathway | 6 | 12.244898 | 1.00E-04 | GSK3B, MAPK8, PIK3CA, JAK2, MAPK14, PIK3CG |
| hsa04071:Sphingolipid signaling pathway | 7 | 14.2857143 | 1.32E-04 | OPRD1, MAPK8, PIK3CA, ROCK1, ROCK2, MAPK14, PIK3CG |
| hsa04722:Neurotrophin signaling pathway | 6 | 12.244898 | 0.00115009 | GSK3B, MAPK8, PIK3CA, MAPKAPK2, MAPK14, PIK3CG |
| hsa04151:PI3K-Akt signaling pathway | 9 | 18.3673469 | 0.00180476 | GSK3B, RPS6KB1, PIK3CA, CCNE1, CDK2, KDR, JAK2, EGFR, PIK3CG |
| hsa04068:FoxO signaling pathway | 6 | 12.244898 | 0.00187949 | MAPK8, PIK3CA, CDK2, MAPK14, EGFR, PIK3CG |
| hsa04668:TNF signaling pathway | 5 | 10.2040816 | 0.0054673 | MAPK8, PIK3CA, MAPK14, PTGS2, PIK3CG |
| hsa04931:Insulin resistance | 5 | 10.2040816 | 0.00565012 | GSK3B, MAPK8, RPS6KB1, PIK3CA, PIK3CG |
| hsa04923:Regulation of lipolysis in adipocytes | 4 | 8.16326531 | 0.00605619 | PIK3CA, ADRB2, PTGS2, PIK3CG |
| hsa04020:Calcium signaling pathway | 6 | 12.244898 | 0.00651968 | DRD1, ADRB2, NOS1, HTR2A, ADRA1A, EGFR |
| hsa04062:Chemokine signaling pathway | 6 | 12.244898 | 0.00764554 | GSK3B, PIK3CA, ROCK1, ROCK2, JAK2, PIK3CG |
| hsa04152:AMPK signaling pathway | 5 | 10.2040816 | 0.00890086 | CCNA2, RPS6KB1, PIK3CA, ADRA1A, PIK3CG |
| hsa04115:p53 signaling pathway | 4 | 8.16326531 | 0.00994501 | CCNE1, CHEK2, CHEK1, CDK2 |
| hsa04015:Rap1 signaling pathway | 6 | 12.244898 | 0.01253466 | PIK3CA, KDR, DRD2, MAPK14, EGFR, PIK3CG |
| hsa04910:Insulin signaling pathway | 5 | 10.2040816 | 0.01318795 | GSK3B, MAPK8, RPS6KB1, PIK3CA, PIK3CG |
| hsa04550:Signaling pathways regulating pluripotency of stem cells | 5 | 10.2040816 | 0.01384372 | GSK3B, PIK3CA, JAK2, MAPK14, PIK3CG |
| hsa04540:Gap junction | 4 | 8.16326531 | 0.02067208 | DRD1, HTR2A, DRD2, EGFR |
| hsa04066:HIF-1 signaling pathway | 4 | 8.16326531 | 0.02594125 | RPS6KB1, PIK3CA, EGFR, PIK3CG |
| hsa04915:Estrogen signaling pathway | 4 | 8.16326531 | 0.02808689 | PIK3CA, OPRM1, EGFR, PIK3CG |
| hsa04660:T cell receptor signaling pathway | 4 | 8.16326531 | 0.02882263 | GSK3B, PIK3CA, MAPK14, PIK3CG |
| hsa04620:Toll-like receptor signaling pathway | 4 | 8.16326531 | 0.03345201 | MAPK8, PIK3CA, MAPK14, PIK3CG |
| hsa04930:Type II diabetes mellitus | 3 | 6.12244898 | 0.04061986 | MAPK8, PIK3CA, PIK3CG |
| hsa04150:mTOR signaling pathway | 3 | 6.12244898 | 0.05708937 | RPS6KB1, PIK3CA, PIK3CG |
| hsa04014:Ras signaling pathway | 5 | 10.2040816 | 0.06325718 | MAPK8, PIK3CA, KDR, EGFR, PIK3CG |
| hsa04662:B cell receptor signaling pathway | 3 | 6.12244898 | 0.07740141 | GSK3B, PIK3CA, PIK3CG |
| hsa04921:Oxytocin signaling pathway | 4 | 8.16326531 | 0.07819254 | ROCK1, ROCK2, PTGS2, EGFR |
| hsa04932:Non-alcoholic fatty liver disease (NAFLD) | 4 | 8.16326531 | 0.07941625 | GSK3B, MAPK8, PIK3CA, PIK3CG |
